# Structural analysis of heme proteins: implications for design and prediction

**DOI:** 10.1186/1472-6807-11-13

**Published:** 2011-03-03

**Authors:** Ting Li, Herbert L Bonkovsky, Jun-tao Guo

**Affiliations:** 1 Cannon Research Center, Carolinas Medical Center, 1000 Blythe Boulevard, Charlotte, NC, 28203, USA; 2 Department of Biology, University of North Carolina at Charlotte, Charlotte, NC, USA; 3 Department of Medicine, University of North Carolina at Chapel Hill, Chapel Hill, NC, USA; 4 Department of Medicine, University of Connecticut Health Center, Farmington, CT, USA; 5 Department of Bioinformatics and Genomics, University of North Carolina at Charlotte, 9201 University City Boulevard, Charlotte, NC 28223, USA

## Abstract

**Background:**

Heme is an essential molecule and plays vital roles in many biological processes. The structural determination of a large number of heme proteins has made it possible to study the detailed chemical and structural properties of heme binding environment. Knowledge of these characteristics can provide valuable guidelines in the design of novel heme proteins and help us predict unknown heme binding proteins.

**Results:**

In this paper, we constructed a non-redundant dataset of 125 heme-binding protein chains and found that these heme proteins encompass at least 31 different structural folds with all-α class as the dominating scaffold. Heme binding pockets are enriched in aromatic and non-polar amino acids with fewer charged residues. The differences between apo and holo forms of heme proteins in terms of the structure and the binding pockets have been investigated. In most cases the proteins undergo small conformational changes upon heme binding. We also examined the CP (cysteine-proline) heme regulatory motifs and demonstrated that the conserved dipeptide has structural implications in protein-heme interactions.

**Conclusions:**

Our analysis revealed that heme binding pockets show special features and that most of the heme proteins undergo small conformational changes after heme binding, suggesting the apo structures can be used for structure-based heme protein prediction and as scaffolds for future heme protein design.

## Background

This year marks the 50^th ^anniversary of the publication of the very first two protein structures, myoglobin and hemoglobin, two prototype heme proteins involved in oxygen storage and transport [1,2]. Heme proteins, or hemoproteins, are a group of proteins carrying heme as the prosthetic group. Heme proteins are ubiquitous in biological systems and exhibit diverse biological activities. These include the classical functions of diatomic gas transportation/storage and electron transfer as exemplified by myoglobin, hemoglobin and cytochrome *c *[3,4]. More recent studies continue to reveal more pleiotropic roles of heme proteins in transcriptional regulation [5,6], ion channel chemosensing [7], circadian clock control [8], and microRNA processing [9].

The identification of human Rev-erb nuclear receptors as heme sensing transcription factors represents an important addition to the heme protein family [10,11]. Rev-erbα (NR1D1) and Rev-erbβ (NR1D2) have been implicated in the regulation of circadian rhythms, lipid and glucose metabolism, and diseases [12-15]. They were previously categorized as orphan receptors with no known physiological ligand. Computational modeling and X-ray crystallization of the ligand binding domain (LBD) of Rev-erbs provided incentives for proposing heme as the *bona fide *ligand. However, the proposal was largely based on the homology between Rev-erb LBD and that of a known heme sensing protein E75, a *Drosophila *nuclear receptor; and the authenticity of heme as a ligand remained elusive at the time due to the lack of unified information on heme binding sites and heme-protein interaction. Therefore detailed analysis and prediction were not possible. Yet the Rev-erb story prompted us to ask: can we predict heme proteins? The worldwide structural genomics projects have produced a large number of new structures with unknown functions or annotated as hypothetical proteins [16,17]. Owing to the ubiquitous and essential nature of heme in life, we hypothesize that some "orphan" structures in Protein Data Bank (PDB) [18] are heme proteins.

**Figure 1:**
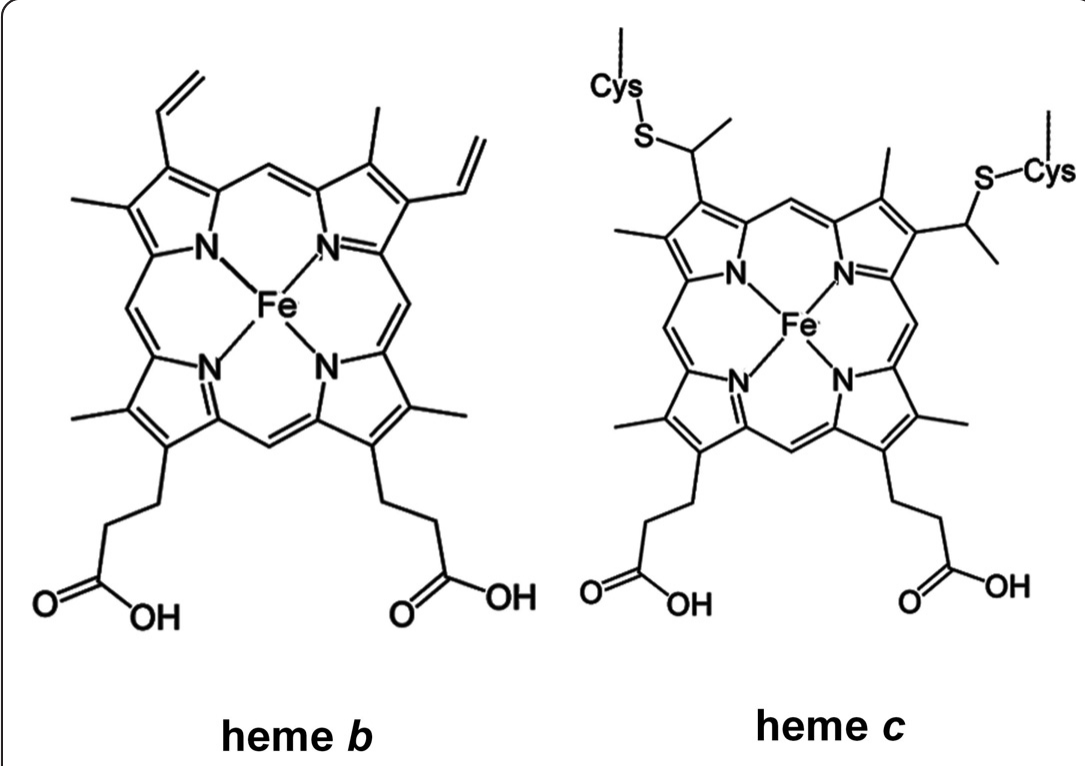
**Chemical structures of heme *b *and heme *c***.

To date, structure-based protein function prediction remains a major challenge in structural bioinformatics. Rational design of heme proteins represents another attractive research front for its potential in the development of advanced biocatalysts and therapeutics [19-25]. Regardless of the purposes, a thorough understanding of protein-ligand interaction is essential. The interactions between heme and its host proteins are complicated. Heme as a prosthetic group can exist in different forms. Among the known forms, heme *b *and heme *c *represent the most common types of heme groups associated with proteins [26]. Heme *b *binds to proteins noncovalently while heme *c *forms covalent bonds between the heme vinyl groups and two cysteine residues of proteins (Figure 1). Previous studies suggested that the functional versatility of heme proteins is delivered not only by the variability of the heme molecules but also the diverse micro-environment of the proteins, the nature of the axial ligands to iron, and the relative solvent accessibility of heme [27,28]. Heme proteins encompass diverse protein fold structures, among which is the well-known globin fold. However as probably the result of convergent evolution, analogous fold structures do not always warrant successful functional inference. For example, the N-terminal domain of RsbR, a protein involved in environmental stress signaling, assumes a globin-fold structure but does not bind to heme [29], highlighting the complexity of heme proteins and the need for detailed analysis of the heme binding surroundings.

As a first step towards a long term goal to develop methodologies for predicting and designing novel heme proteins, a field of interest with great potential in medicine and green energy [27,30], we set out to investigate the common characteristics of heme binding sites and the conformational differences between apo (without heme) and holo heme proteins, aiming at consolidating and synthesizing a large body of experimental data and extracting useful information and novel integrative insights.

We take into consideration two key questions crucial to the structure-function paradigm of heme proteins. The first concerns the structural implications of the heme-interactive sequence motifs. CXXCH represents the classic type-*c *heme binding motif in which the two vinyl groups of heme form covalent bonds with two cysteine residues in proteins [27,28]. Recently, a heme regulatory motif CP (for cysteine-proline dipeptide) has received increasing attention [31-35]. But up to the present the functional importance of this CP heme sensing or regulatory motif has been studied only through mutational experiments on a limited number of proteins. It is still not clear from a structural point of view how the CP motif is involved in regulation of heme binding as has been established for the CXXCH heme *c *motif.

The second question concerns the structural environment or the physiochemical features of the heme binding pockets. Of particular importance is the conformational difference between the apo and holo forms of heme proteins since, in most cases, only apo structures will be available for prediction. Even though the global and local conformational changes induced by ligand binding in general have been surveyed by a number of studies [36-39], such systematic studies on heme proteins have not been reported. In this study, we compiled a non-redundant dataset of apo-holo pairs to examine the conformational and pocket changes in heme proteins after heme binding.

The diversity and conservation of interactions between heme and proteins have been analyzed previously by Schneider *et al*. [27]. However they used a redundant dataset with 68 type-*b *heme proteins (based on 60% sequence identity cutoff) due largely to the limited availability of heme protein structures [27,40]. A very recent study performed analysis on a smaller dataset of 34 heme proteins, each of which represents one CATH homologous family or a SCOP family [41]. There are seven different heme groups in the 34 heme proteins with heme *b *and heme *c *as the dominant forms [41]. Here we performed structural analysis on a larger, non-redundant dataset of heme proteins containing heme *b *and/or *c *types. Heme proteins are found in at least 31 different structural folds in all the four major classes based on SCOP classifications [42], attesting to the diversity and complexity of heme-protein interactions. The heme binding pockets are enriched in aromatic amino acids and relatively depleted with respect to the charged residues, glutamic acid, aspartic acid, and lysine. We also found that the CP motif has structural implications in heme-protein interactions.

## Methods

### Datasets

Two non-redundant datasets were generated in this study. The first dataset, containing 125 heme-binding protein chains, was used for analysis of heme binding environment. This set was culled from protein structures in the Protein Data Bank (PDB, November 24, 2009) [18] with HEM (for heme *b*) or HEC (for heme *c*) as ligands with the following criteria: experimental method = X-ray crystallography, maximum resolution = 3 Å, and maximum R-value = 0.3. The protein chains that interact with heme molecules (described in next section "*Analysis of heme interacting residues*") were selected, and a non-redundant set of 125 heme-binding protein chains was generated using PISCES [43] with a sequence identity cutoff of 25% (Additional file 1, Table S1). The second dataset has 5596 protein chains in which each pair of protein chains has less than 25% sequence identity and each structure has a resolution of 2.5 Å or better and an R-factor of 0.3 or better. This set was used for calculating background frequencies of amino acids, secondary structure types, and relative solvent accessibility. The sequences for the protein chains derived from the PDB "SEQRES" records may have cloning and expression artifacts such as His-tags at the N- or C-terminus and some of the protein chains have missing residues [44,45]. To avoid such artifacts and incomplete sequences, the amino acid frequencies were calculated using the full-length protein sequences through mapping PDB chains to Uniprot entries with PDBSWS [46].

### Analysis of heme interacting residues

A residue is considered as a heme axial ligand if the distance between the nitrogen, sulfur or oxygen of the residue and the heme iron is within 3 Å. Residues having heavy atoms within 4.5 Å of any non-hydrogen atoms of the heme molecule are identified as heme interacting amino acids. A protein chain is considered as heme binding if it has residue(s) as axial ligand(s) to the heme iron or has at least ten residue interactions with the heme molecule. DSSP was used to assign each residue to one of three secondary structure states, helix, strand, and coil [47]. Following the widely used convention, H (α-helix), G (3_10_-helix) and I (π-helix) from DSSP are classified as helix type while E (extended strand) and B (residue in isolated-bridge) states are classified as strand type. All the other states from DSSP are considered as coils. The relative solvent accessibility was calculated by dividing the absolute value of exposed area from DSSP over the maximum accessibility of each residue [48]. We employ a three-state classification for relative solvent accessibility: buried (≤7%), intermediate (>7% and ≤37%), and exposed (>37%), as described previously [49].

### Structural comparisons between apo and holo heme proteins

To maximize the number of possible apo-holo heme protein pairs, each of the heme protein chains was first compared with all the non-heme protein chains derived from PISCES pdbaaent file using BLAST [50]. There are a number of ligands that are similar to heme *b *or *c *in PDB, so structures with these heme-like ligands are not considered as apo proteins for our apo-holo comparisons. Based on HIC-Up keyword search using heme and porphyrin [51] and SuperLigands ligand structure similarity search [52], we identified 55 heme-like ligands in PDB (Additional file 1, Table S2). The highly similar apo-holo heme protein pairs (cutoffs set at 90% sequence identity and 95% sequence alignment overlap) were then culled to generate a list of 15 non-redundant apo-holo pairs using PISCES with a sequence identity cutoff of 25% [43]. Five of the 15 apo proteins that contain other non-heme ligands in the heme-binding pockets were removed from the list as they are not truly "apo" forms with respect to the heme binding sites. The structural differences were evaluated with two structure alignment programs, FAST [53] and CE [54] for structure comparisons. The similarity/difference between two structures is measured by the RMSD (root mean square deviation) of the Cα atoms of aligned residues. The pocket/cavity was predicted using the CASTp server (Computed Atlas of Surface Topography of proteins)[55]. To compare the shape of the pockets, Rvs, the ratio between the volume and the surface area is used[56].

## Results and Discussion

### Non-redundant dataset of heme binding proteins

**Figure 2:**
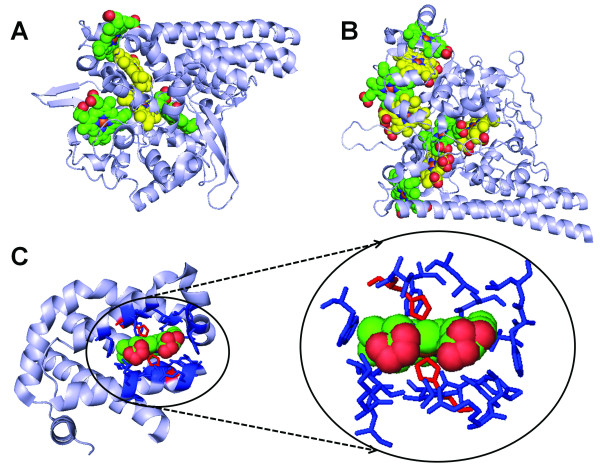
**Examples of three-dimensional structure of multi-heme proteins and identification of heme-binding environment**. (A) Cytochrome c nitrite reductase of *Wolinella succinogenes *(PDB chain: 1FS7A) with 5 heme *b *molecules; (B) *Thioalkalivibrio nitratireducens *cytochrome c nitrite reductase (PDB chain: 3F29A) with 8 heme *c *molecules; (C) globin domain of globin-coupled sensor in *Geobacter sulfurreducens *(PDB chain: 2W31A). The red sticks are axial ligands of the heme iron and the blue sticks represent other heme interacting residues. For better visualization, the neighboring heme residues in A and B are colored yellow and green respectively. The heme molecules are shown as spacefill. The images were generated using Pymol http://www.pymol.org.

There are 1998 and 113 PDB entries containing ligand HEM (heme type-*b*) and HEC (heme type-*c*) respectively with resolutions of 3Å or better as of November 24, 2009 [18]. Among these entries, 10 (1BE3, 1BGY, 1FGJ, 1GWS, 1PP9, 1PPJ, 1S56, 1S61, 2A06, and 3H1J) contain both heme type *b *and *c*. *In toto *4272 protein chains were identified as heme interacting protein chains as described in Methods. A non-redundant dataset of 125 protein chains (114 heme-*b *and 11 heme-*c*, Additional file 1, Table S1) were generated using PISCES with a sequence identity cutoff of 25%[43]. Eighty-two percent of these protein chains contain only one heme molecule while the number of heme molecules in the remaining protein chains ranges from 2 to 8 (Additional file 1, Table S1). Two examples of multi-heme protein chains, 1FS7A with 5 type *b *and 3F29A with 8 type *c *heme molecules, are shown in (Figure 2A &2B).

The dataset of heme binding proteins includes a wide variety of protein folds. A total of 86 protein chains (~69% of the dataset) have SCOP annotations (based on release 1.75 and Pre-SCOP) and belong to 31 distinct structural folds in all four major classes (Table 1) [42]. The dataset is dominated by proteins in the all-α class, making up 64% (55 of 86) of the total. The top 4 folds, Globin-like (a.1), Cytochrome P450 (a.104), Cytochrome *c *(a.3), and Multi-heme cytochromes (a.138) represent the well-known heme binding proteins that have been investigated extensively (Table 1).

**Table 1:** SCOP fold classes of the 125 heme binding protein chains in the non-redundant dataset

SCOP Fold	# of Chains	SCOP Fold Name
a.104	9	Cytochrome P450
a.1	14	Globin-like
a.123	1	Nuclear receptor ligand-binding domain
a.126	1	Serum albumin-like
a.132	2	Heme oxygenase-like
a.138	8	Multiheme cytochromes
a.24	2	Four-helical up-and-down bundle
a.266	2	Indolic compounds 2,3-dioxygenase-like
a.3	9	Cytochrome c
a.39	1	EF Hand-like
a.45	1	GST C-terminal domain-like
a.93	5	Heme-dependent peroxidases
b.1	4	Immunoglobulin-like beta-sandwich
b.2	1	Common fold of diphtheria toxin/transcription factors...
b.60	2	Lipocalins
b.66	1	4-bladed beta-propeller
b.82	1	Double-stranded beta-helix
c.150	1	EreA/ChaN-like
c.47	1	Thioredoxin fold
c.79	1	Tryptophan synthase β subunit-like PLP-dependent enzymes
c.92	1	Chelatase-like
d.110	1	Profilin-like
d.120	1	Cytochrome b5-like heme/steroid binding domain
d.151	1	DNase I-like
d.174	1	Nitric oxide (NO) synthase oxygenase domain
d.278	2	Ligand-binding domain in NO signalling & Golgi transport
d.58	3	Ferredoxin-like
e.5	2	Heme-dependent catalase-like
e.62	1	Heme iron utilization protein-like
f.21	5	Heme-binding four-helical bundle
f.24	1	Cytochrome c oxidase subunit I-like

**Total: 31**	**86**	

### Structural environment of the heme binding pockets

**Figure 3:**
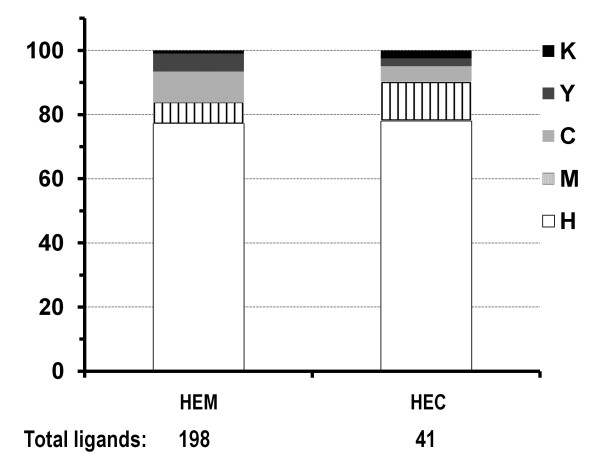
**Distribution of the axial ligands for heme *b *(HEM) and heme *c *(HEC)**.

To investigate the structural environment of heme binding pockets, we identified both residues that make coordinate bonds with the heme iron and the ones that interact with the heme porphyrin structure (Figure 2C and Methods). Out of the 125 heme binding protein chains, only 2PBJA and 3HCNA do not have residues identified as axial ligands to heme iron though both have extensive interactions with heme; instead other small molecules, such as glutathione (GSH) in 2PBJA (microsomal prostaglandin E synthase)[57] and imidazole (IMD) in 3HCNA (human ferrochelatase) [58] form coordinate bonds with heme iron. Five different amino acids (H, M, C, Y, K) are found to serve as axial ligands to the heme iron with histidine as the dominant residue (~80%) in both heme *b *and heme *c *types (Figure 3). Heme *b *utilizes more cysteine residues while heme *c *has slightly more methionine residues as axial ligands. It should be pointed out that there are only 41 residues as heme *c *ligands. Therefore the percentages of non-histidine ligands may have a relatively large change with a slight increase or decrease of ligand numbers due to the small dataset.

The conserved interactions between protein residues and heme were previously studied by calculating either the frequencies of residues that are in *van der Waals *contact with heme for each fold class of *b*-type heme proteins [27] or by calculating the mean number per binding site [41]. Smith *et al *also applied normalized amino acid profiles to assess the composition and conservation of heme binding sites [41]. Here we explored the residue preferences in the heme binding pockets through calculating the relative frequencies of heme binding residues in our non-redundant dataset. The relative frequency of each amino acid is normalized to its background frequency.

Normally, the background frequencies used for comparisons are calculated from a non-redundant protein dataset. However, due to the dominant presence of all-α folds, it is not clear whether the residue distribution in heme proteins is different from that in other proteins. Therefore we first compared the residue distributions between non-redundant heme proteins and non-redundant all proteins. To avoid issues with missing residues and cloning artifacts (His-tags etc.) associated with PDB sequences, we used native full-length protein sequences to calculate residue compositions by mapping the PDB chains to Uniprot entries with PDBSWS [46]. The relative residue frequencies between heme proteins and all proteins show that heme proteins tend to contain more alanine, phenylalanine, histidine, methionine, and tryptophan residues and fewer cysteine, aspartic acid, isoleucine, lysine, asparagine, and serine residues (Additional file 2, Figure S1). Statistical analysis (χ^2^) revealed a significant difference between these two frequency profiles (data not shown). In order to have a meaningful description of the enrichment or deficiency of residues in the heme interacting environment, we used the background frequencies from the non-redundant set of heme proteins as references.

**Figure 4:**
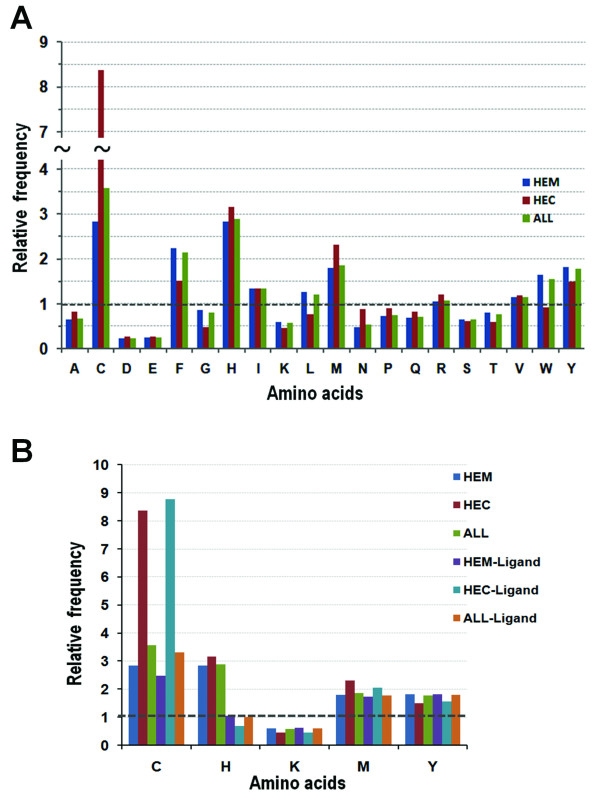
**Relative frequency of the heme interacting amino acids**. (A) Relative frequency of residues in heme *b *(HEM), heme *c *(HEC), and heme *b *and *c *(ALL); (B) the relative frequency of the 5 residues with or without them as axial heme ligands.

The top five residues with high relative frequencies are cysteine (C), histidine (H), phenylalanine (F), methionine (M), and tyrosine (Y) (Figure 4A). Because four of the top five (C, H, M, and Y) can serve as axial ligands to heme iron (Figure 3), we removed axial ligands from the dataset and recalculated the relative frequencies. Figure 4B shows that the level of histidine decreases to the background level, suggesting the enrichment of histidine is essentially due to the large number of heme histidine ligands. The other four residues, on the other hand, have almost the same relative frequencies with or without ligand residues (Figure 4B). In heme *c *proteins, the occurrence of cysteine residues is extremely high with an eight fold enrichment compared to the background distribution. This is not surprising as the classic CXXCH binding motif, in which the histidine serve as ligand and the cysteine residues form covalent thioether bonds with the heme vinyl groups, has dominant presence in heme *c *proteins[28].

Consistent with earlier reports, the aromatic residues (phenylalanine, tyrosine, and tryptophan) play important roles in protein-heme interactions through stacking interactions with the porphyrin[27,41]. One exception is tryptophan in heme *c *proteins, which showed a similar level of occurrences compared to the background (Figure 4A). Leucine, isoleucine, and valine, which make hydrophobic interactions with the heme ring structure, are slightly increased over the background frequencies. The residues with the fewest occurrences, aspartic acid, glutamic acid, and lysine are charged residues, suggesting the heme binding pocket is mainly a hydrophobic environment. In contrast, arginine, a positively charged residue that has been considered a major player in anchoring the heme propionates, has a much higher occurrence than other charged amino acids and shows a similar (HEM) or slightly higher (HEC) level of frequency to the background (Figure 4A) [27].

**Figure 5:**
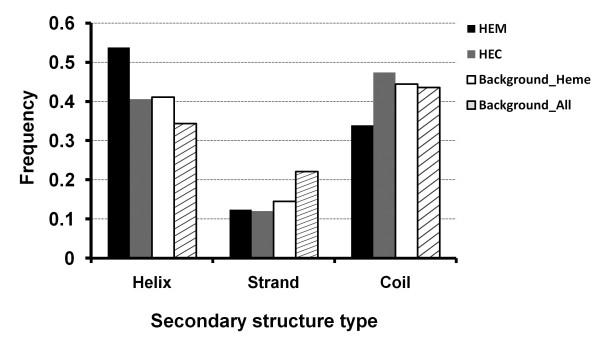
**Frequencies of secondary structure types for heme interacting residues**.

The secondary structure types for heme interacting residues are shown in Figure 5. There are more helical and less coil types in proteins with heme *b *no matter what dataset (heme proteins or all proteins) is used as a reference. Therefore the difference is not due to the large number of all-α proteins in the dataset. As for heme interacting residues in heme *c*, they have similar distribution to the background (Figure 5). Based on our 3-category classification of relative solvent accessibility [49], the heme interacting residues are less likely to be exposed. The buried residues are comparable to the background distribution. About 20% increase is observed in the intermediate category (Additional file 2, Figure S2).

### Heme binding sequence motifs

**Figure 6:**
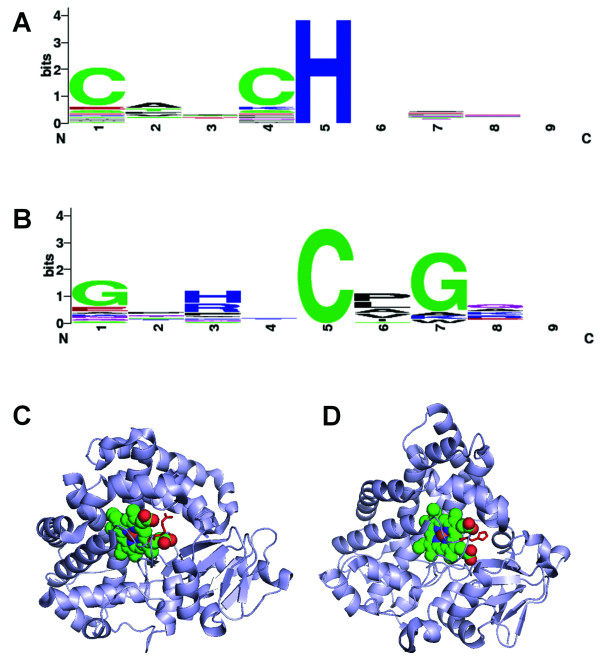
**Sequence motifs surrounding the axial ligands**. (A) Sequence logo from 32 heme *c *proteins with histidine as axial ligand shows the classic CXXCH heme *c *binding motif; (B) Sequence logo from 18 heme *b *proteins with cysteine as axial ligand. The sequence logos were created with WebLogo [74]; (C) Arginine-334 (red sticks) of 1N97A interacts with heme propionates (red spheres); and (D) Interactions between histidine-353 (red sticks) of 1GWIA and heme propionate groups (red spheres).

To investigate possible sequence motifs involved in heme binding, the flanking sequences with four residues on each side of heme axial ligands were collected and aligned. The non-redundant dataset has 34 heme *c *ligands, 32 of which have histidine as axial ligands. The alignment of these sequences shows the classic CXXCH heme *c *binding motif [4,28] (Figure 6A).

Another motif worthy of note, **G**X[HR]X**C**[PLAV]**G**, comes from the heme *b *proteins with cysteine as axial ligands (Figure 6B). The motif represents the classic CYP signature heme binding motif FXXGXXCXG in bacteria, plant, and mammalian cytochrome P450 s [59-61]. At the -4 and +2 positions (with ligand cysteine as reference position) are small amino acids (glycine) while the -2 position prefers a positively charged amino acid such as histidine or arginine. These positively charged residues interact electronically with the negatively charged heme propionates (Figure 6C and 6D). The small glycine residue at the -4 position may provide the flexibility needed for positioning the positively charged residues close to heme propionate groups. The +1 position is dominated by proline and hydrophobic amino acids, leucine, alanine, valine and isoleucine. Six of the eighteen cases have proline right after the axial ligand cysteine, reminiscent of the dipeptide CP motif being implicated in heme sensing and regulation [31-35,62]. While the importance of CP motif has been studied through deletion or site-directed mutation experiments in several important proteins, including transcription repressor Bach1[63], iron regulatory protein 2 (IRP2) [31], circadian factor period 2 (Per2) [34] and δ-aminolevulinic acid synthase (ALAS) [33], the possible role of the CP motif in heme interaction from a structural point of view remains unclear as the structures for most of these proteins with such CP motifs are unknown.

**Figure 7:**
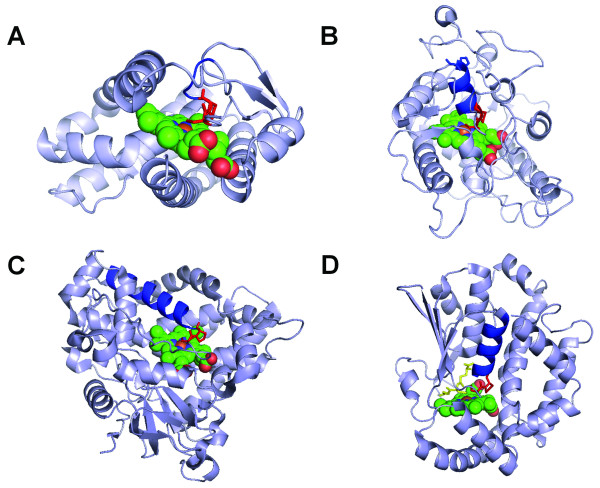
**Three-dimensional structures of heme proteins with "CP" motifs**. (A) 3CQVA; (B) 2CIWA; (C) 1GIWA; and (D) 2PBJA. The CP dipeptides are shown as red sticks. The immediate downstream structures of the CP dipeptides are shown in blue.

All the six CP dipeptides that have direct physical interactions with heme exhibit similar structural roles with the cysteines serving as ligands to the heme iron and the proline residue introducing a bend for the downstream structures, mainly α-helices, to steer them away from the heme face (Figure 7B and 7C). A seventh protein chain, 2PBJA, contains a CP where the proline shows highly similar structural implication, whereas the cysteine residue interacts with heme but not as a ligand. Instead, the presence of a glutathione molecule (GSH), which forms a coordination bond with the heme iron, seems to push the cysteine slightly away from the axial ligand position (5.25 Å from heme iron) [57]. Considering the conformation in the proline-bend structure and the small distance between cysteine and heme iron, it is likely that the cysteine could serve as a heme ligand if GSH is not present in the structure. Interestingly, a closer examination of the structural conformation downstream of the proline residue in 2CIWA (cloroperoxidase), 3CQVA (Rev-erb), and 2PBJA (microsomal prostaglandin E synthase), which have the CP heme motifs with conserved proline, indicates nearly perpendicular orientation to the heme plane (Figure 7A, 7B and 7D). In contrast, in the P450 family where the proline residue is less conserved, with leucine, isoleucine, and methionine also found at the position of proline as shown in the motif logo (Figure 6B), the α-helices following the proline residue are in parallel with the heme plane (Figure 7C). The difference suggests a different structural role for the proline in conserved CP dipeptides from that in the less-conserved CP dipeptides, more specifically at the proline position.

CP dipeptides have also been implicated in indirect interaction with heme. Ragsdale and colleagues reported a novel role for CP motifs in heme oxygenase 2 (HMOX-2) as a thiol/disulfide redox switch that localizes outside the heme-binding pocket [62,64,65], therefore regulating heme-protein interaction via sensing redox status in the environment. There are a total of twenty-nine CP dipeptides in our dataset. Less than a quarter of them (in 7 protein chains including 2PBJA) show physical interactions with heme molecules. It would be impractical at this point to predict the functional role of the remaining CP dipeptides in heme-protein interaction, mainly due to the limited sample size and the lack of structural details on heme pocket-CP interaction. Here we made use of statistical analysis to indirectly assess the functional relevance of CP dipeptides in heme interaction. The rationale behind the assay is that, if CP dipeptides are important heme signatures for heme interaction, the expected occurrences of CP dipeptides in hemoproteins should be higher compared to control population. We found no statistically significant difference between the presence of CP dipeptides in heme proteins and non-heme proteins (data not shown), suggesting other yet to be identified factors may exist to help determine the role CP dipeptides play in heme binding [31]. It should be noted that we do not exclude the possibility that in the control sample there exist unknown hemoproteins; however for them to significantly affect the frequency of CP signals there would have to be a considerably large fraction of the control proteins being analyzed to be heme-interacting, which we anticipate as less likely.

### Structure comparison between apo and holo heme proteins

**Figure 8:**
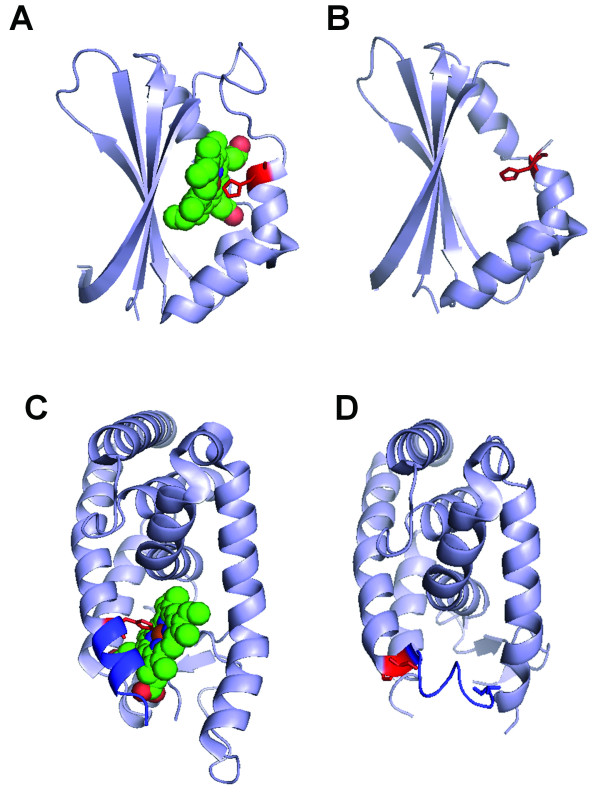
**Structural comparison of apo-holo heme protein pairs**. (A,B) 2ZDOA-1XBWD; (C,D) 3CQVA-2V7CA.

An interesting question related to structure-based heme binding protein design and prediction is the degree of global conformational transition and the local changes of the heme-binding pocket upon heme binding. We collected 446 heme protein chains (after removing heme protein chains with at least 90% sequence identity) and compared their sequences with the protein chains without heme or heme-like ligands (Additional file 1, Table S2). One hundred seventy-nine heme protein chains are found to have apo structures with high sequence similarity and coverage. After removing redundant apo/holo pairs with a 25% sequence identity cutoff and proteins with non-heme or non-heme-like ligands occupying the heme binding pocket, the final dataset consists of 10 apo-holo protein pairs. Table 2 shows that 9 out of 10 proteins undergo very small global conformational changes after heme binding with RMSDs of 1.03Å or less. For example the 2ZDOA-1XBWD pair (iron-regulated surface determinant IsdG from *Staphylococcus aureus*) has an RMSD of 0.59 Å. In the absence of heme, the protein assumes the same conformation as the holo protein with heme (Figure 8A, B). Even the side chain positions of the histidine ligand are similar. The one with relatively large conformational changes is Rev-erb (3CQVA-2V7CA). Without heme the C-terminal helix (residues 568-576) moves towards the heme pocket with His568 (heme-binding ligand) facing away from the binding pocket (Figure 8C, D) [66].

**Table 2:** Comparisons between apo and holo heme protein structures

holo-chain	volume (Å^3^)	surface area (Å^2^)	Rvs^a^	apo-chain	volume (Å^3^)	surface area (Å^2^)	Rvs^a^	sequence identity%	RMSD (Å)
**1KBIA**	458.1	432	1.060	**1SZFB**	1039.3	708.3	1.468	99	0.40
**1N45A**	1634.7	1139.3	1.435	**1S8CD**	845.1	634.4	1.332	100	0.86
**1N5UA**	*	*	*	**3CX9A**	*	*	*	100	0.60
**2ITFA**	721.7	521	1.385	**2ITEB**	803.4	551	1.457	100	0.60
**2NWBA**	*	*	*	**1ZEEB**	*	*	*	96	0.75
**2OFRX**	1211.1	857.4	1.413	**2OFMX**	783.3	642.7	1,219	100	1.03
**2R7AA**	1362.6	963.4	1.414	**2RG7D**	1558.1	1019.6	1.528	100	0.91
**2ZDOA**	808.7	553.9	1.460	**1XBWD**	766.9	544.2	1.409	99	0.59
**3CQVA**	1638.3	1099	1.490	**2V7CA**	537.1	436.2	1.232	94	2.32
**3EMMA**	399	383.1	1.042	**2A13A**	910.8	635.2	1.434	100	0.53

^a ^Rvs: ratio between volume and surface area

* Data not shown because CASTp predicts a very large cavity, covering several pockets.

**Figure 9:**
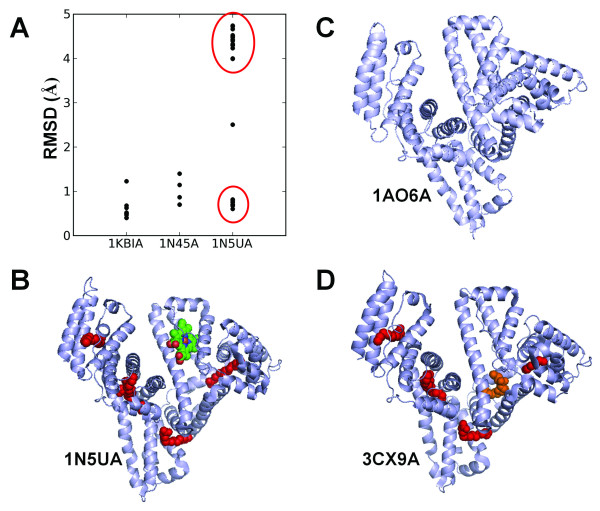
**Examples of heme proteins with multiple apo structures**. (A) RMSD distribution of apo structure of three heme protein chains, 1KBIA, 1N45A, and 1N5UA. The 28 apo structures of 1N5UA form two clusters (red ovals). (B) Structure of 1N5UA with one heme and five myristic acid molecules (MYR, red spacefill). (C) Structure of 1AO6A with no ligands. (D) Structure of 3CX9A with five myristic acid molecules (MYR, red spacefill) and one LPX ligand (orange spacefill).

Three of the ten heme proteins in Table 2 have multiple known apo structures. 1KBIA (flavin-binding domain of Baker's yeast flavocytochrome b_2_), 1N45A (human heme oxygenase-1), and 1N5UA (human serum albumin) have 9, 3, and 28 apo structures respectively (with at least 99% sequence identity, Additional file 1, Table S3). Because proteins are inherently dynamic and conformational selection has been considered as a major mechanism for biomolecular recognition [67-69], we checked the conformational differences between each of the apo structures and the holo structures. Figure 9A shows the RMSD (Cα atoms of aligned residues) values of the apo-holo structural differences. The RMSDs are generally less than 1Å for 1KBIA and 1N45A. On the contrary, apo structures of 1N5UA form two clusters. Members of one cluster with 12 apo structures have RMSDs around 0.8Å while the other contains 15 apo structures with RMSDs ranging from 4 to 5Å. Through manual inspection, we found that the differences are caused by the numbers of non-heme ligands in structures. In addition to heme, 1N5UA also has 5 myristic acid (MYR) molecules (Figure 9B). The apo structures with higher RMSDs either do not have ligands (Figure 9C) or have only one or two non-MYR ligands. For example, 1E7AA and 2BX8B have 2 PFL and 1 AZQ respectively. On the other hand, apo structures with MYR ligands in similar positions as those in 1N5UA generally have smaller RMSDs (Figure 9D). Therefore, under similar environment, there are relatively small structural differences between holo and apo heme protein structures.

It should be noted that the above comparisons are based on heme proteins that have stable apo structures solved through X-ray crystallography. For some proteins, as in the case of hemoglobin, the absence of ligand(s) can increase the flexibility and cause partial unfolding of the protein structure, making it difficult for structure determination [70,71]. Furthermore, intrinsically disordered or unstructured regions are considered to be responsible for many important cellular functions such as ligand binding [72,73]. However the existence of such flexible apo structures would not interfere with our goal in structure-based heme protein prediction as we aim to take the existing apo structures in PDB as inputs [18].

Other features useful for comparing apo-holo heme proteins are the pocket size and shape. Due to different heme binding modes (partially exposed or fully embedded, Additional file 2 Figure S3) and the difficulty in identifying the exact heme binding pocket from existing automatic programs, the sizes of heme binding pockets vary from small (~400 Å^3^) to very large (over 2000 Å^3^) (Table 2). In addition, the changes in absolute pocket volumes after heme binding are variable. Small changes are seen in 2ITFA-2ITEB, 2R7AA-2RG7 D, and 2ZDOA-1XBWD. Other pairs exhibited significant changes in volume despite the minimal conformational change (Table 2). To take the shape into consideration we calculated the Rvs value (the ratio of pocket volume over the pocket surface area) of each pocket. Most of the apo or holo proteins have Rvs values around 1.4. To further investigate whether the binding pocket can be used as one of the characteristics for heme protein prediction, we compared the Rvs distributions between heme binding pockets and pockets in non-heme proteins (proteins that don't have heme ligand(s) and are not homologous to heme proteins) with similar sizes ranging from 350 to 2000Å^3^. The Rvs of heme binding pockets has a narrow distribution whereas the Rvs from similar pocket sizes of non-heme proteins has a wide spread with a long right tail (Additional file 2, Figure S4-A). We also investigated the distribution of Rvs normalized to a sphere shape as introduced by Sonavane and Chakrabarti [56]. A similar trend was found (Additional file 2, Figure S4-B). It should be pointed out that, even though unknown heme proteins may be included in the non-heme dataset, many non-heme proteins share similar pocket characteristics.

## Conclusion

In this study, we surveyed the known heme protein structures for the purpose of structure-based heme protein prediction and novel heme protein design. We first compiled a non-redundant dataset of 125 heme (type *b *and *c*) binding protein chains that encompass a large number of protein structural folds, reflecting the diversified roles of heme proteins. Structural analysis revealed that the residues interacting with heme are mainly non-polar, especially aromatic amino acids, providing a hydrophobic environment for the heme ring structure. We also investigated the possible structural roles of CP motifs that are implicated in the regulation of heme binding and have received much attention recently. While the CP dipeptide is not as strong a signature for heme binding as the classic CXXCH heme *c *binding motif, the proline in the heme-interacting CP dipeptides assume important structural roles when CP is conserved and the cysteine functions as an axial ligand with heme iron. Indirect interaction between CP motifs and heme binding has also been reported in HMOX-2 protein, in which CP dipeptides form thiol/disulfide redox switch away from the heme binding pocket [62,64], suggesting the heterogeneity of CP-heme interactions.

Comparisons between the apo and holo heme proteins indicate that most of the heme proteins undergo small conformational changes after heme binding, suggesting the apo structure can be used for structure-based heme protein prediction and as a scaffold for heme protein design. In addition our analysis on the heme binding pockets showed that despite the different sizes, the Rvs values of heme binding pockets are confined in a small range, whereas the data from non-heme binding proteins spread over a large range. We will apply the results from this study to investigate if any of the hypothetical proteins in PDB are potential heme proteins through computational prediction and experimental validations in the near future.

## List of abbreviations used

Å: angstrom; CP: cysteine-proline; LBD: lipid binding domain; SCOP: structural classification of proteins; PDB: protein data bank; RMSD: root mean square deviation; Rvs: ratio of volume over area.

## Authors' contributions

TL and JTG conceived the project and wrote the manuscript. JTG wrote the programs and performed the structural analysis. HLB participated the discussion of the project and was involved the revision of the manuscript. All authors read and approved the final manuscript.

## Supplementary Material

Additional file 1 **Datasets used in structural analysis of heme proteins**. Table S1: a list of 125 non-redundant heme-binding protein chains; Table S2: heme and heme-like ligands in PDB; Table S3: heme proteins with multiple apo structures Click here for file

Additional file 2 **Comparative analysis of heme-binding proteins and non-heme proteins in terms of amino acid frequency, relative solvent accessibility, and Rvs (ratio between volume and area)**. Figure S1: relative frequencies of amino acids in non-redundant heme proteins; Figure S2: frequencies of relative solvent accessibility for heme interacting residues; Figure S3: heme binding pockets in EMMA (A) and 3CQVA (B); Figure S4: distribution of (A) Rvs (ratio between volume and area) and (B) nRvs (normalized Rvs) between heme binding pockets and non-heme binding pockets. Click here for file

## References

[B1] Kendrew JC Dickerson RE Strandberg BE Hart RG Davies DR Phillips DC Shore VC Structure of myoglobin: A three-dimensional Fourier synthesis at 2 A. resolution Nature 1960 185 4711 422 427 10.1038/185422a0 18990802

[B2] Perutz MF Rossmann MG Cullis AF Muirhead H Will G North AC Structure of haemoglobin: a three-dimensional Fourier synthesis at 5.5-A. resolution, obtained by X-ray analysis Nature 1960 185 4711 416 422 10.1038/185416a0 18990801

[B3] Poulos TL The Janus nature of heme Nat Prod Rep 2007 24 3 504 510 10.1039/b604195g 17534526

[B4] Paoli M Marles-Wright J Smith A Structure-function relationships in heme-proteins DNA Cell Biol 2002 21 4 271 280 10.1089/104454902753759690 12042067

[B5] Sun J Hoshino H Takaku K Nakajima O Muto A Suzuki H Tashiro S Takahashi S Shibahara S Alam J Taketo MM Yamamoto M Igarashi K Hemoprotein Bach1 regulates enhancer availability of heme oxygenase-1 gene The EMBO journal 2002 21 19 5216 5224 10.1093/emboj/cdf516 12356737 PMC129038

[B6] Zenke-Kawasaki Y Dohi Y Katoh Y Ikura T Ikura M Asahara T Tokunaga F Iwai K Igarashi K Heme induces ubiquitination and degradation of the transcription factor Bach1 Molecular and cellular biology 2007 27 19 6962 6971 10.1128/MCB.02415-06 17682061 PMC2099246

[B7] Tang XD Xu R Reynolds MF Garcia ML Heinemann SH Hoshi T Haem can bind to and inhibit mammalian calcium-dependent Slo1 BK channels Nature 2003 425 6957 531 535 10.1038/nature02003 14523450

[B8] Kaasik K Lee CC Reciprocal regulation of haem biosynthesis and the circadian clock in mammals Nature 2004 430 6998 467 471 10.1038/nature02724 15269772

[B9] Faller M Matsunaga M Yin S Loo JA Guo F Heme is involved in microRNA processing Nature structural & molecular biology 2007 14 1 23 29 10.1038/nsmb1182 17159994

[B10] Raghuram S Stayrook KR Huang P Rogers PM Nosie AK McClure DB Burris LL Khorasanizadeh S Burris TP Rastinejad F Identification of heme as the ligand for the orphan nuclear receptors REV-ERBalpha and REV-ERBbeta Nature structural & molecular biology 2007 14 12 1207 1213 10.1038/nsmb1344 PMC2743565 18037887

[B11] Yin L Wu N Curtin JC Qatanani M Szwergold NR Reid RA Waitt GM Parks DJ Pearce KH Wisely GB Lazar MA Rev-erbalpha, a heme sensor that coordinates metabolic and circadian pathways Science 2007 318 5857 1786 1789 10.1126/science.1150179 18006707

[B12] Coste H Rodriguez JC Orphan nuclear hormone receptor Rev-erbalpha regulates the human apolipoprotein CIII promoter J Biol Chem 2002 277 30 27120 27129 10.1074/jbc.M203421200 12021280

[B13] Migita H Morser J Kawai K Rev-erbalpha upregulates NF-kappaB-responsive genes in vascular smooth muscle cells FEBS letters 2004 561 1-3 69 74 10.1016/S0014-5793(04)00118-8 15013753

[B14] Preitner N Damiola F Lopez-Molina L Zakany J Duboule D Albrecht U Schibler U The orphan nuclear receptor REV-ERBalpha controls circadian transcription within the positive limb of the mammalian circadian oscillator Cell 2002 110 2 251 260 10.1016/S0092-8674(02)00825-5 12150932

[B15] Yang X Lamia KA Evans RM Nuclear receptors, metabolism, and the circadian clock Cold Spring Harb Symp Quant Biol 2007 72 387 394 10.1101/sqb.2007.72.058 18419296

[B16] Watson JD Sanderson S Ezersky A Savchenko A Edwards A Orengo C Joachimiak A Laskowski RA Thornton JM Towards fully automated structure-based function prediction in structural genomics: a case study Journal of molecular biology 2007 367 5 1511 1522 10.1016/j.jmb.2007.01.063 17316683 PMC2566530

[B17] Pazos F Sternberg MJ Automated prediction of protein function and detection of functional sites from structure Proceedings of the National Academy of Sciences of the United States of America 2004 101 41 14754 14759 10.1073/pnas.0404569101 15456910 PMC522026

[B18] Berman HM Westbrook J Feng Z Gilliland G Bhat TN Weissig H Shindyalov IN Bourne PE The Protein Data Bank Nucleic acids research 2000 28 1 235 242 10.1093/nar/28.1.235 10592235 PMC102472

[B19] Reedy CJ Gibney BR Heme protein assemblies Chem Rev 2004 104 2 617 649 10.1021/cr0206115 14871137

[B20] Isogai Y Ishida M Design of a novel heme protein with a non-heme globin scaffold Biochemistry 2009 48 34 8136 8142 10.1021/bi900518q 19601582

[B21] Koder RL Anderson JL Solomon LA Reddy KS Moser CC Dutton PL Design and engineering of an O(2) transport protein Nature 2009 458 7236 305 309 10.1038/nature07841 19295603 PMC3539743

[B22] Lu Y Yeung N Sieracki N Marshall NM Design of functional metalloproteins Nature 2009 460 7257 855 862 10.1038/nature08304 19675646 PMC2770889

[B23] Lin YW Yeung N Gao YG Miner KD Tian S Robinson H Lu Y Roles of glutamates and metal ions in a rationally designed nitric oxide reductase based on myoglobin Proceedings of the National Academy of Sciences of the United States of America 2010 107 19 8581 8586 10.1073/pnas.1000526107 20421510 PMC2889330

[B24] Choma CT Lear JD Nelson MJ Dutton PL Robertson DE Degrado WF Design of a heme-binding 4-helix bundle Journal of the American Chemical Society 1994 116 3 856 865 10.1021/ja00082a005

[B25] Robertson DE Farid RS Moser CC Urbauer JL Mulholland SE Pidikiti R Lear JD Wand AJ DeGrado WF Dutton PL Design and synthesis of multi-haem proteins Nature 1994 368 6470 425 432 10.1038/368425a0 8133888

[B26] Reedy CJ Elvekrog MM Gibney BR Development of a heme protein structure-electrochemical function database Nucleic acids research 2008 36 Database D307 313 17933771 10.1093/nar/gkm814 PMC2238922

[B27] Schneider S Marles-Wright J Sharp KH Paoli M Diversity and conservation of interactions for binding heme in b-type heme proteins Nat Prod Rep 2007 24 3 621 630 10.1039/b604186h 17534534

[B28] Bowman SE Bren KL The chemistry and biochemistry of heme c: functional bases for covalent attachment Nat Prod Rep 2008 25 6 1118 1130 10.1039/b717196j 19030605 PMC2654777

[B29] Murray JW Delumeau O Lewis RJ Structure of a nonheme globin in environmental stress signaling Proceedings of the National Academy of Sciences of the United States of America 2005 102 48 17320 17325 10.1073/pnas.0506599102 16301540 PMC1297668

[B30] Razeghifard R Wallace BB Pace RJ Wydrzynski T Creating functional artificial proteins Curr Protein Pept Sci 2007 8 1 3 18 10.2174/138920307779941479 17305556

[B31] Igarashi J Murase M Iizuka A Pichierri F Martinkova M Shimizu T Elucidation of the heme binding site of heme-regulated eukaryotic initiation factor 2alpha kinase and the role of the regulatory motif in heme sensing by spectroscopic and catalytic studies of mutant proteins J Biol Chem 2008 283 27 18782 18791 10.1074/jbc.M801400200 18450746

[B32] Ishikawa H Kato M Hori H Ishimori K Kirisako T Tokunaga F Iwai K Involvement of heme regulatory motif in heme-mediated ubiquitination and degradation of IRP2 Molecular cell 2005 19 2 171 181 10.1016/j.molcel.2005.05.027 16039587

[B33] Lathrop JT Timko MP Regulation by heme of mitochondrial protein transport through a conserved amino acid motif Science 1993 259 5094 522 525 10.1126/science.8424176 8424176

[B34] Yang J Kim KD Lucas A Drahos KE Santos CS Mury SP Capelluto DG Finkielstein CV A novel heme-regulatory motif mediates heme-dependent degradation of the circadian factor period 2 Molecular and cellular biology 2008 28 15 4697 4711 10.1128/MCB.00236-08 18505821 PMC2493356

[B35] Zhang L Guarente L Heme binds to a short sequence that serves a regulatory function in diverse proteins The EMBO journal 1995 14 2 313 320 7835342 10.1002/j.1460-2075.1995.tb07005.x PMC398085

[B36] Brylinski M Skolnick J What is the relationship between the global structures of apo and holo proteins? Proteins 2008 70 2 363 377 10.1002/prot.21510 17680687

[B37] Karthikeyan S Zhou Q Osterman AL Zhang H Ligand binding-induced conformational changes in riboflavin kinase: structural basis for the ordered mechanism Biochemistry 2003 42 43 12532 12538 10.1021/bi035450t 14580199

[B38] Najmanovich R Kuttner J Sobolev V Edelman M Side-chain flexibility in proteins upon ligand binding Proteins 2000 39 3 261 268 10.1002/(SICI)1097-0134(20000515)39:3<261::AID-PROT90>3.0.CO;2-4 10737948

[B39] Zavodszky MI Kuhn LA Side-chain flexibility in protein-ligand binding: the minimal rotation hypothesis Protein Sci 2005 14 4 1104 1114 10.1110/ps.041153605 15772311 PMC2253453

[B40] Dessailly BH Nair R Jaroszewski L Fajardo JE Kouranov A Lee D Fiser A Godzik A Rost B Orengo C PSI-2: structural genomics to cover protein domain family space Structure 2009 17 6 869 881 10.1016/j.str.2009.03.015 19523904 PMC2920419

[B41] Smith LJ Kahraman A Thornton JM Heme proteins--diversity in structural characteristics, function, and folding Proteins 2010 78 10 2349 2368 10.1002/prot.22747 20544970

[B42] Murzin AG Brenner SE Hubbard T Chothia C SCOP: a structural classification of proteins database for the investigation of sequences and structures Journal of molecular biology 1995 247 4 536 540 7723011 10.1006/jmbi.1995.0159

[B43] Wang G Dunbrack RL Jr PISCES: a protein sequence culling server Bioinformatics 2003 19 12 1589 1591 10.1093/bioinformatics/btg224 12912846

[B44] Carson M Johnson DH McDonald H Brouillette C Delucas LJ His-tag impact on structure Acta Crystallogr D Biol Crystallogr 2007 63 Pt 3 295 301 10.1107/S0907444906052024 17327666

[B45] Kim R Guo JT Systematic analysis of short internal indels and their impact on protein folding BMC Struct Biol 2010 10 1 24 10.1186/1472-6807-10-24 20684774 PMC2924343

[B46] Martin AC Mapping PDB chains to UniProtKB entries Bioinformatics 2005 21 23 4297 4301 10.1093/bioinformatics/bti694 16188924

[B47] Kabsch W Sander C Dictionary of protein secondary structure: pattern recognition of hydrogen-bonded and geometrical features Biopolymers 1983 22 12 2577 2637 10.1002/bip.360221211 6667333

[B48] Miller S Janin J Lesk AM Chothia C Interior and surface of monomeric proteins Journal of molecular biology 1987 196 3 641 656 10.1016/0022-2836(87)90038-6 3681970

[B49] Kim D Xu D Guo JT Ellrott K Xu Y PROSPECT II: protein structure prediction program for genome-scale applications Protein engineering 2003 16 9 641 650 10.1093/protein/gzg081 14560049

[B50] Altschul SF Gish W Miller W Myers EW Lipman DJ Basic local alignment search tool Journal of molecular biology 1990 215 3 403 410 2231712 10.1016/S0022-2836(05)80360-2

[B51] Kleywegt GJ Crystallographic refinement of ligand complexes Acta Crystallogr D Biol Crystallogr 2007 63 Pt 1 94 100 17164531 10.1107/S0907444906022657 PMC2483469

[B52] Michalsky E Dunkel M Goede A Preissner R SuperLigands-a database of ligand structures derived from the Protein Data Bank BMC bioinformatics 2005 6 122 10.1186/1471-2105-6-122 15943884 PMC1173082

[B53] Zhu J Weng Z FAST: a novel protein structure alignment algorithm Proteins 2005 58 3 618 627 10.1002/prot.20331 15609341

[B54] Shindyalov IN Bourne PE Protein structure alignment by incremental combinatorial extension (CE) of the optimal path Protein engineering 1998 11 9 739 747 10.1093/protein/11.9.739 9796821

[B55] Dundas J Ouyang Z Tseng J Binkowski A Turpaz Y Liang J CASTp: computed atlas of surface topography of proteins with structural and topographical mapping of functionally annotated residues Nucleic acids research 2006 34 Web Server W116 118 10.1093/nar/gkl282 16844972 PMC1538779

[B56] Sonavane S Chakrabarti P Cavities and atomic packing in protein structures and interfaces PLoS Comput Biol 2008 4 9 e1000188 10.1371/journal.pcbi.1000188 19005575 PMC2582456

[B57] Yamada T Takusagawa F PGH2 degradation pathway catalyzed by GSH-heme complex bound microsomal prostaglandin E2 synthase type 2: the first example of a dual-function enzyme Biochemistry 2007 46 28 8414 8424 10.1021/bi700605m 17585783

[B58] Medlock AE Carter M Dailey TA Dailey HA Lanzilotta WN Product release rather than chelation determines metal specificity for ferrochelatase Journal of molecular biology 2009 393 2 308 319 10.1016/j.jmb.2009.08.042 19703464 PMC2771925

[B59] Nelson DR Cytochrome P450 and the individuality of species Archives of biochemistry and biophysics 1999 369 1 1 10 10.1006/abbi.1999.1352 10462435

[B60] Chapple C Molecular-genetic analysis of plant cytochrome P450-dependent monooxygenases Annu Rev Plant Physiol Plant Mol Biol 1998 49 311 343 10.1146/annurev.arplant.49.1.311 15012237

[B61] Otyepka M Skopalik J Anzenbacherova E Anzenbacher P What common structural features and variations of mammalian P450 s are known to date? Biochimica et biophysica acta 2007 1770 3 376 389 17069978 10.1016/j.bbagen.2006.09.013

[B62] Yi L Jenkins PM Leichert LI Jakob U Martens JR Ragsdale SW Heme regulatory motifs in heme oxygenase-2 form a thiol/disulfide redox switch that responds to the cellular redox state J Biol Chem 2009 284 31 20556 20561 10.1074/jbc.M109.015651 19473966 PMC2742820

[B63] Ogawa K Sun J Taketani S Nakajima O Nishitani C Sassa S Hayashi N Yamamoto M Shibahara S Fujita H Igarashi K Heme mediates derepression of Maf recognition element through direct binding to transcription repressor Bach1 The EMBO journal 2001 20 11 2835 2843 10.1093/emboj/20.11.2835 11387216 PMC125477

[B64] Yi L Morgan JT Ragsdale SW Identification of a thiol/disulfide redox switch in the human BK channel that controls its affinity for heme and CO J Biol Chem 2010 285 26 20117 20127 10.1074/jbc.M110.116483 20427280 PMC2888424

[B65] Yi L Ragsdale SW Evidence that the heme regulatory motifs in heme oxygenase-2 serve as a thiol/disulfide redox switch regulating heme binding J Biol Chem 2007 282 29 21056 21067 10.1074/jbc.M700664200 17540772 PMC3957417

[B66] Pardee KI Xu X Reinking J Schuetz A Dong A Liu S Zhang R Tiefenbach J Lajoie G Plotnikov AN Botchkarev A Krause HM Edwards A The structural basis of gas-responsive transcription by the human nuclear hormone receptor REV-ERBbeta PLoS Biol 2009 7 2 e43 10.1371/journal.pbio.1000043 19243223 PMC2652392

[B67] Tsai CJ del Sol A Nussinov R Allostery: absence of a change in shape does not imply that allostery is not at play Journal of molecular biology 2008 378 1 1 11 10.1016/j.jmb.2008.02.034 18353365 PMC2684958

[B68] Okazaki K Takada S Dynamic energy landscape view of coupled binding and protein conformational change: induced-fit versus population-shift mechanisms Proceedings of the National Academy of Sciences of the United States of America 2008 105 32 11182 11187 10.1073/pnas.0802524105 18678900 PMC2516237

[B69] Boehr DD Nussinov R Wright PE The role of dynamic conformational ensembles in biomolecular recognition Nat Chem Biol 2009 5 11 789 796 10.1038/nchembio.232 19841628 PMC2916928

[B70] Leutzinger Y Beychok S Kinetics and mechanism of heme-induced refolding of human alpha-globin Proceedings of the National Academy of Sciences of the United States of America 1981 78 2 780 784 10.1073/pnas.78.2.780 6940147 PMC319886

[B71] Culbertson DS Olson JS Role of heme in the unfolding and assembly of myoglobin Biochemistry 2010 49 29 6052 6063 10.1021/bi1006942 20540498 PMC2912444

[B72] Dunker AK Brown CJ Obradovic Z Identification and functions of usefully disordered proteins Unfolded Proteins 2002 62 25 49 full_text 10.1016/s0065-3233(02)62004-2 12418100

[B73] Dyson HJ Wright PE Intrinsically unstructured proteins and their functions Nat Rev Mol Cell Biol 2005 6 3 197 208 10.1038/nrm1589 15738986

[B74] Crooks GE Hon G Chandonia JM Brenner SE WebLogo: a sequence logo generator Genome research 2004 14 6 1188 1190 10.1101/gr.849004 15173120 PMC419797

