# Supplementary material for: Structural analysis of heme proteins: implications for design and prediction
Source: BMC Struct Biol. 2011 Mar 3;11:13. doi: 10.1186/1472-6807-11-13 (PMC3059290; doi:10.1186/1472-6807-11-13)
Supplement: Additional file 1 — Datasets used in structural analysis of heme proteins. Table S1: a list of 125 non-redundant heme-binding protein chains; Table S2: heme and heme-like ligands in PDB; Table S3: heme proteins with multiple apo structures [file 1472-6807-11-13-S1.PDF]

Table S1: A list of 125 non-redundant heme-binding protein chains

Protein ID	Length (AA)	Ligand	Resolution	R-factor	# of heme
1ASHA	150	HEM	2.150	0.18	1
1C2RA	116	HEM	2.500	0.17	1
1CTJA	89	HEM	1.100	0.14	1
1D0CA	444	HEM	1.650	0.21	1
1D2VC	466	HEM	1.750	0.24	1
1DLWA	116	HEM	1.540	0.13	1
1ECAA	136	HEM	1.400	0.18	1
1FS7A	485	HEM	1.600	0.18	5
1FT5A	211	HEM	1.600	0.19	4
1GVHA	396	HEM	2.190	0.19	1
1GWEA	503	HEM	0.880	0.09	1
1GWIA	411	HEM	1.920	0.21	1
1GWUA	309	HEM	1.310	0.17	1
1H97A	147	HEM	1.170	0.12	1
1IW0A	215	HEM	1.400	0.17	1
1IZOA	417	HEM	2.100	0.25	1
1J0PA	108	HEM	0.910	0.11	4
1J77A	209	HEM	1.500	0.23	1
1JBQA	435	HEM	2.600	0.26	1
1JF3A	147	HEM	1.400	0.19	1
1JNIA	123	HEM	1.250	0.16	2
1KQFC	217	HEM	1.600	0.18	2
1M1QA	91	HEM	0.970	0.14	4
1MJ4A	82	HEM	1.200	0.12	1
1N40A	396	HEM	1.060	0.13	1
1N5UA	585	HEM	1.900	0.23	1
1N97A	389	HEM	1.800	0.21	1
1OR4A	178	HEM	2.150	0.20	1
1PBYA	489	HEM	1.700	0.19	2
1PL3A	186	HEM	1.900	0.17	1
1PO5A	476	HEM	1.600	0.22	1
1Q1FA	151	HEM	1.500	0.22	1
1QHUA	460	HEM	2.300	0.20	1
1RWJA	82	HEM	1.700	0.20	3
1TU9A	134	HEM	1.200	0.16	1
1U55A	188	HEM	1.770	0.23	1
1U5UA	374	HEM	2.000	0.21	1
1V9YA	167	HEM	1.320	0.16	1
1X8QA	184	HEM	0.850	0.10	1
1XMEA	568	HEM	2.300	0.22	1
1Y5IC	225	HEM	1.900	0.18	2
1YIQA	689	HEM	2.200	0.17	1

1Z9NA	177	HEM	1.500	0.16	1
256BA	106	HEM	1.400	0.16	1
2BKMA	128	HEM	1.500	0.17	1
2BS2C	256	HEM	1.780	0.23	2
2CIBA	455	HEM	1.500	0.20	1
2CIWA	299	HEM	1.150	0.15	1
2CZSA	80	HEM	1.500	0.22	2
2D0TA	406	HEM	2.300	0.24	1
2D3QA	442	HEM	2.800	0.27	1
2E3BA	344	HEM	1.300	0.15	1
2FMYA	220	HEM	2.200	0.21	1
2FWTA	125	HEM	1.850	0.15	2
2G5GX	268	HEM	1.900	0.21	1
2GDMA	153	HEM	1.700	0.16	1
2H88C	140	HEM	1.740	0.18	1
2H88D	103	HEM	1.740	0.18	1
2HQ2A	354	HEM	1.450	0.17	1
2IIZA	312	HEM	2.300	0.21	1
2IJ2A	470	HEM	1.200	0.15	1
2IMQX	282	HEM	1.300	0.18	1
2ITFA	127	HEM	1.900	0.17	2
2IVFC	214	HEM	1.880	0.15	1
2J8WA	129	HEM	1.290	0.15	1
2LHBA	150	HEM	2.000	0.14	1
2NRLA	147	HEM	0.910	0.15	1
2NW8A	306	HEM	1.600	0.17	1
2NWBA	400	HEM	2.400	0.22	1
2O09A	189	HEM	2.100	0.19	1
2O6PA	161	HEM	1.500	0.20	2
2OYYA	76	HEM	2.500	0.24	1
2PBJA	290	HEM	2.800	0.24	1
2Q7AA	152	HEM	2.100	0.17	2
2Q8PA	260	HEM	1.950	0.20	1
2R7AA	256	HEM	2.050	0.21	1
2V7KA	361	HEM	1.700	0.18	1
2VE3A	444	HEM	2.100	0.23	1
2VEBA	195	HEM	1.300	0.16	1
2VV6A	119	HEM	1.500	0.18	1
2VXHA	251	HEM	2.100	0.22	1
2W31A	162	HEM	1.500	0.17	1
2W3GA	153	HEM	1.400	0.19	1
2WDQC	129	HEM	2.400	0.17	1
2WDQD	115	HEM	2.400	0.17	1
2WIYA	394	HEM	1.490	0.15	1
2WJNC	336	HEM	1.860	0.17	4
2Z6FA	131	HEM	1.900	0.20	1

2Z8AA	146	HEM	1.060	0.14	1
2ZDOA	109	HEM	1.800	0.20	1
2ZS0D	155	HEM	1.600	0.17	1
2ZWUA	415	HEM	1.300	0.17	1
3A16A	373	HEM	1.600	0.25	1
3B47A	134	HEM	2.000	0.25	1
3B98A	475	HEM	2.080	0.23	1
3BXUA	71	HEM	1.350	0.16	3
3BZ1E	84	HEM	2.900	0.25	1
3BZ1F	45	HEM	2.900	0.25	1
3CQVA	199	HEM	1.900	0.20	1
3CSLA	865	HEM	2.700	0.24	1
3CX5C	385	HEM	1.900	0.24	2
3D1KA	142	HEM	1.250	0.17	1
3DANA	473	HEM	1.800	0.18	1
3DR9A	137	HEM	1.260	0.17	1
3E2CA	158	HEM	1.800	0.20	1
3E2OA	294	HEM	1.060	0.17	1
3E4WA	320	HEM	1.800	0.20	1
3ELLA	184	HEM	1.700	0.17	1
3EMMA	166	HEM	1.360	0.17	1
3EQMA	503	HEM	2.900	0.22	1
3H8TA	191	HEM	1.800	0.16	1
3HCNA	359	HEM	1.600	0.21	1
3IVYA	433	HEM	1.350	0.16	1
3PGHA	587	HEM	2.500	0.24	1
1E29A	135	HEC	1.210	0.15	1
1E2WA	251	HEC	1.600	0.20	1
1GU2A	124	HEC	1.190	0.15	1
1H21A	247	HEC	2.500	0.20	4
1H32A	261	HEC	1.500	0.20	3
1M70A	190	HEC	1.250	0.16	2
1YNRA	80	HEC	2.000	0.17	1
2C1VA	338	HEC	1.200	0.17	2
2JE3A	186	HEC	1.800	0.20	1
3CP5A	124	HEC	1.240	0.13	1
3F29A	25	HEC	2.000	0.15	8

--Protein chains with more than one heme molecules are shaded.

Table S2. Heme and heme-like ligands in PDB

ID	Formula	Name
1CP	$C_{36} H_{44} N_4 O_8$	coproporphyrin I
1FH	$C_{40} H_{38}$ FE $N_4 O_4$	12-phenylheme
2FH	$C_{40} H_{36}$ FE $N_4 O_4$	2-phenylheme
6HE	$C_{32} H_{30}$ FE $N_4 O_2$	6-methy-6-depropionatehemin
7HE	$C_{32} H_{30}$ FE $N_4 O_2$	7-methyl-7-depropionatehemin
BCL	$C_{55} H_{74}$ MG N_4	bacteriochlorophyll a
BLA	$C_{33} H_{34} N_4 O_6$	biliverdin ix alpha
CCH	$C_{33} H_{30}$ FE $N_4 O_5$	clorocruoro hem
CL2	$C_{55} H_{72}$ MG $N_4 O_5$	beta chlorophyll a
CLN	$C_{34} H_{32}$ FE $N_4 O_4$ S	sulfur substituted protoporphyrin ix
COH	$C_{34} H_{32}$ CO $N_4 O_4$	protoporphyrin ix containing co
CP3	$C_{36} H_{44} N_4 O_8$	coproporphyrin iii
CPO	$C_{35} H_{53}$ FE $N_6 O_{13}$	coprogen
DDH	$C_{34} H_{32}$ FE $N_4 O_6$	diacetyldeuteroheme
DEU	$C_{30} H_{28}$ CO $N_4 O_4$	co(iii)-(deuteroporphyrin ix)
DHE	$C_{34} H_{32}$ FE $N_4 O_{10}$	heme d
FCI	$C_{28} H_{44}$ FE $N_9 O_{13}$	ferricrocin-iron
FDD	$C_{32} H_{32}$ FE $N_4 O_4$	fe(iii) 2,4-dimethyl deuteroporphyrin ix
FDE	$C_{30} H_{28}$ FE $N_4 O_4$	fe(iii) deuteroporphyrin ix
FEC	$C_{36} H_{36}$ FE $N_4 O_8$	fe-coproporphyrin iii
FMI	$C_{34} H_{36}$ FE $N_4 O_5$	fe-(4-mesoporphyrinone)-r-isomer
H02	$C_{35} H_{42} N_4 O_4$	N-methyl protoporphyrin ix 2,4-disulfonic acid
HAS	$C_{54} H_{64}$ FE $N_4 O_6$	heme-as
HCO	$C_{34} H_{34}$ FE $N_4 O_5$	2-acetyl-protoporphyrin ix
HDD	$C_{34} H_{32}$ FE $N_4 O_5$	cis-heme d hydroxychlorin gamma-spirolactone
HDM	$C_{36} H_{36}$ FE $N_4 O_4$	dimethyl propionate ester heme
HE5	$C_{32} H_{34}$ N4 O_4 ZN	zinc(ii)-deuteroporphyrin dimethylester
HE6	$C_{28} H_{24}$ FE $N_4 O_4$	6,7-dicarboxyl-1,2,3,4,5,8-hexamethylhemin
HEA	$C_{49} H_{56}$ FE $N_4 O_6$	heme-a
HEB	$C_{34} H_{34}$ FE $N_4 O_4$	heme b/c
HEC	$C_{34} H_{34}$ FE $N_4 O_4$	heme c
HEG	$C_{34} H_{32}$ MG $N_4 O_4$	protoporphyrin ix containing mg
HEM	$C_{34} H_{32}$ FE $N_4 O_4$	protoporphyrin ix containing fe
HEO	$C_{49} H_{58}$ FE $N_4 O_5$	heme o
HES	$C_{34} H_{34} N_4 O_4$ ZN	zinc substituted heme c
HEV	$C_{36} H_{32}$ FE $N_4 O_4$	1,3-dedimethyl-1,3-divinyl heme
HFM	$C_{33} H_{32}$ FE $N_4 O_5$	2-formyl-protoporphryn ix
HKL	$C_{34} H_{34}$ Fe $N_4 O_3$	Fe(III) pyropheophorbide-a methyl ester
HIF	$C_{34} H_{36}$ FE $N_4 O_5$	fe(iii)-(4-mesoporphyrinone)
HME	$C_{34} H_{36}$ FE $N_4 O_4$	porphycene containing fe
HNI	$C_{34} H_{32} N_4$ NI O_4	protoporphyrin ix containing ni(ii)
MMP	$C_{35} H_{40} N_4 O_4$	n-methylmesoporphyrin
MNH	$C_{34} H_{32}$ MN $N_4 O_4$	manganese protoporphyrin ix
MNR	$C_{34} H_{32}$ MN $N_4 O_4$	protoporphyrin ix containing mn
MP1	$C_{35} H_{39}$ CU $N_4 O_4$	n-methylmesoporphyrin w/ copper
PC3	$C_{36} H_{36}$ CO $N_4 O_8$	coproporphyrin i containing co(iii)
PCU	$C_{44} H_{36} N_8$ CU	cu(ii)meso(4-n-tetramethylpyridyl) porphyrin
PFC	$C_{34} H_{48}$ FE $N_9 O_{12}$	phenylferricrocin-iron
PHO	$C_{55} H_{74} N_4 O_5$	pheophytin a
PNI	$C_{44} H_{36} N_8$ NI	tetra(n-methyl-pyridyl) porphyrin-nickel
POR	$C_{20} H_{12}$ FE N_4	porphyrin fe(iii)
PP9	$C_{34} H_{34} N_4 O_4$	protoporphyrin ix
SRM	$C_{42} H_{42}$ FE $N_4 O_{16}$	siroheme
UP3	$C_{40} H_{38} N_4 O_{16}$	porphyrin-2,7,12,18-tetrayl]tetrapropanoic acid
VER	$C_{30} H_{27}$ FE $N_4 O_3$	iron-octaethylporphyrin
ZEM	$C_{34} H_{32} N_4 O_5$ ZN	zn(ii)-(20-oxo-protoporphyrin ix)
ZNH	$C_{34} H_{32} N_4 O_4$ ZN	protoporphyrin ix containing zn

Table S3. Heme proteins with multiple apo structures

Holo protein	Apo proteins
**1KBIB**	1FCBB, 1KBIB, 1LCOB, 1LDCB, 1LTDB, 1SZEB, 1SZFB, 1SZGB, and 2OZ0B
**1N45A**	1NI6B, 1S8CB, and 1S8CD
**1N5UA**	1AO6A, 1BJ5A,1BM0B, 1E78A, 1E7AA, 1E7BA, 1E7CA, 1E7FA, 1EFHA, 1GNIA, 1H9ZA, 1HK1A, 1HK3A, 1HK5A, 1TF0A, 1UORA, 2BX8B, 2BXAB, 2BXEB, 2BXFB, 2BXGB, 2BXHB, 2BXKA, 2BXMA, 2BXOA, 2I30A, 3B9LA, and 3CX9A
