# Supplementary material for: Structural analysis of heme proteins: implications for design and prediction
Source: BMC Struct Biol. 2011 Mar 3;11:13. doi: 10.1186/1472-6807-11-13 (PMC3059290; doi:10.1186/1472-6807-11-13)

Figure S1. Relative frequencies of amino acids in non-redundant heme proteins. The relative frequencies are normalized with the amino acid frequencies from non-redundant all proteins.

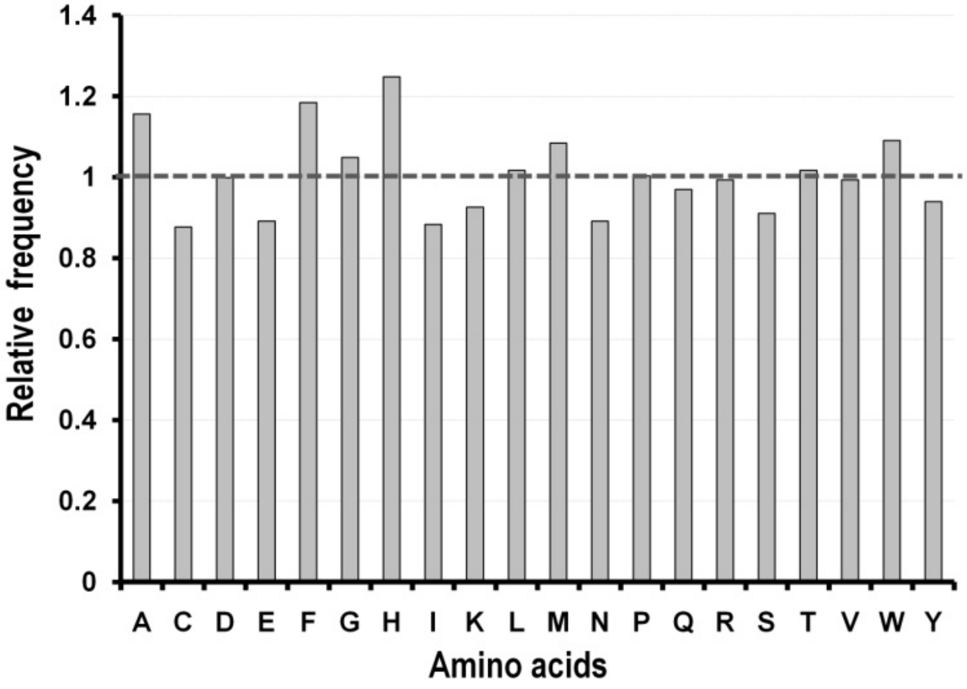

Figure S2. Frequencies of relative solvent accessibility for heme interacting residues

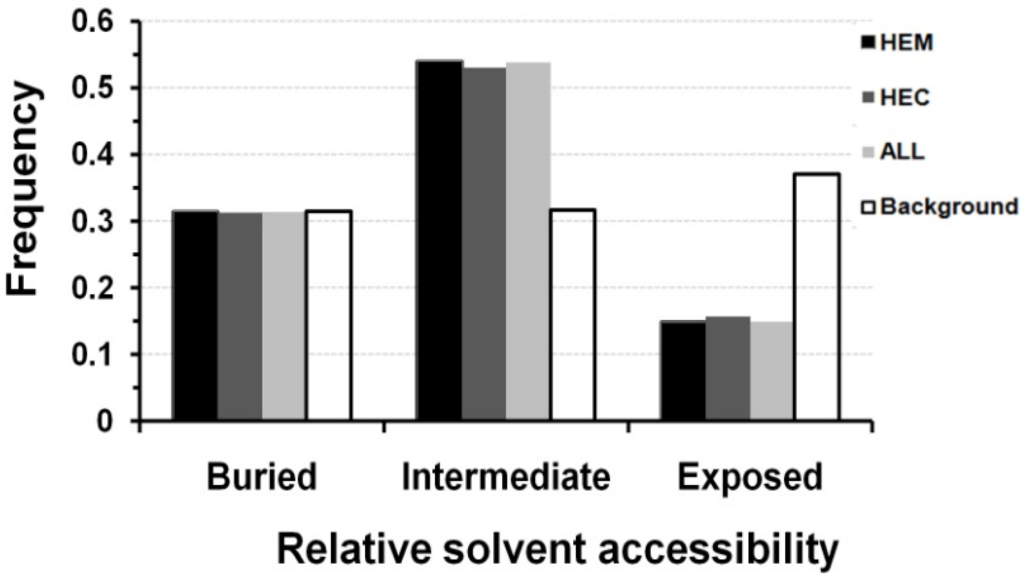

Figure S3. Heme binding pockets in EMMA (A) and 3CQVA (B) based on CASTp calculation [54]. Heme is shown as CPK spacefill. The surface of each pocket is covered with green balls.

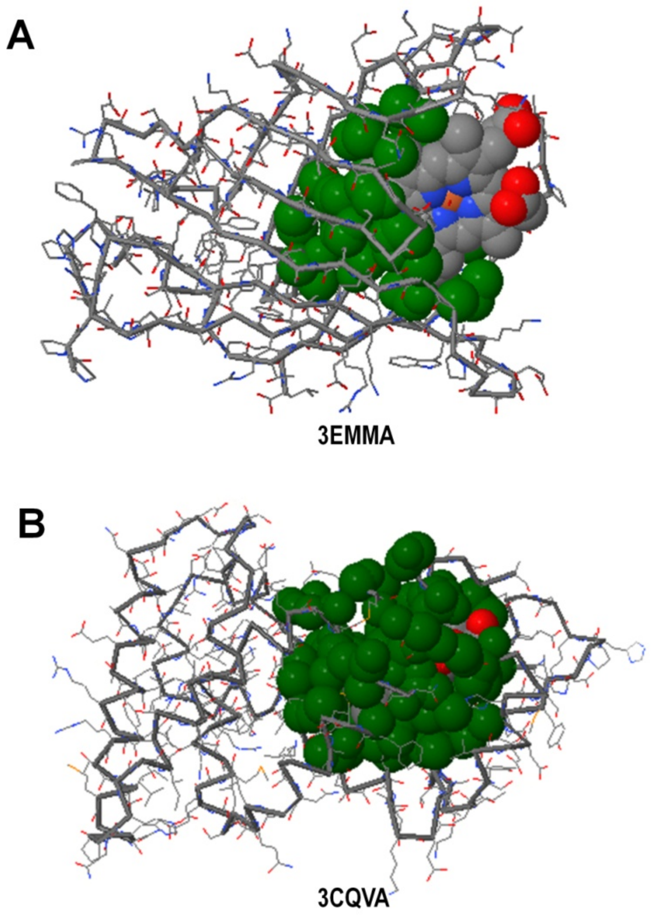

Figure S4. Distribution of (A) Rvs (ratio between volume and area) and (B) nRvs (normalized Rvs) between heme binding pockets and non-heme binding pockets.

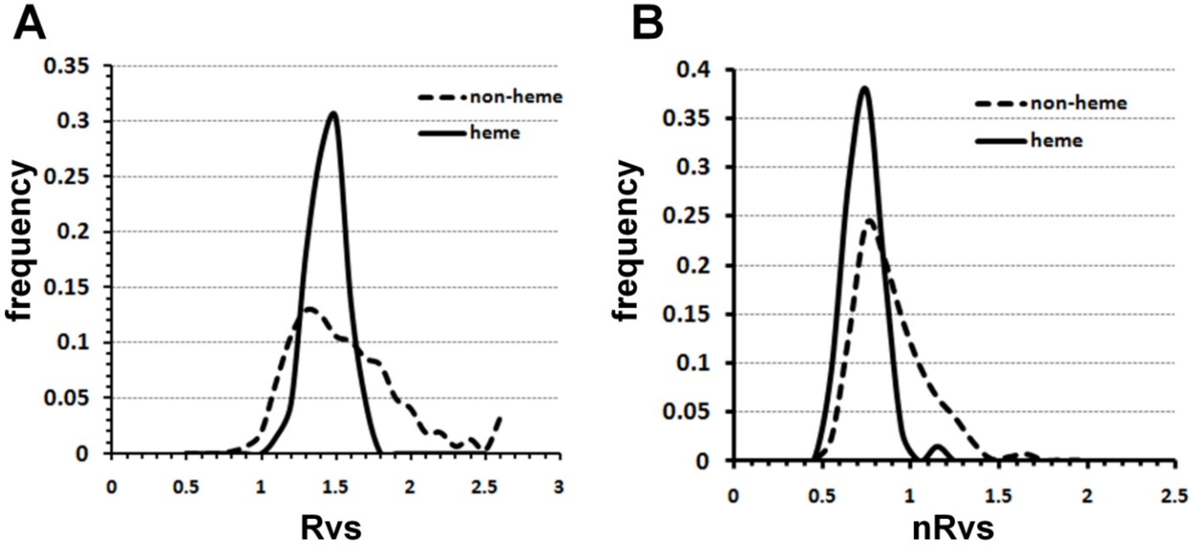

Supplement: Additional file 2 — Comparative analysis of heme-binding proteins and non-heme proteins in terms of amino acid frequency, relative solvent accessibility, and Rvs (ratio between volume and area). Figure S1: relative frequencies of amino acids in non-redundant heme proteins; Figure S2: frequencies of relative solvent accessibility for heme interacting residues; Figure S3: heme binding pockets in EMMA (A) and 3CQVA (B); Figure S4: distribution of (A) Rvs (ratio between volume and area) and (B) nRvs (normalized Rvs) between heme binding pockets and non-heme binding pockets. [file 1472-6807-11-13-S2.PDF]
